# Factors Associated With Urgent-Start Peritoneal Dialysis Catheter Complications in ESRD

**DOI:** 10.1016/j.ekir.2020.07.025

**Published:** 2020-07-26

**Authors:** José L. Hernández-Castillo, Joana Balderas-Juárez, Omar Jiménez-Zarazúa, Karen Guerrero-Toriz, Michelle P. Loeza-Uribe, Erika K. Tenorio-Aguirre, Jesús G. Mendoza-García, Jaime D. Mondragón

**Affiliations:** 1 Facultad de Medicina, Unidad de Posgrado, Universidad Nacional Autónoma de México, Mexico City, Mexico; 2 Department of Internal Medicine, Hospital General Dr. Manuel Gea González, Mexico City, Mexico; 3 Department of Medicine and Nutrition, Universidad de Guanajuato, Guanajuato, Mexico; 4 Department of Internal Medicine, Hospital General León, León, Mexico; 5 Department of Neurology, University of Groningen, University Medical Center Groningen, Groningen, The Netherlands; 6 Alzheimer Center Groningen, Department of Neurology, University of Groningen, University Medical Center Groningen, Groningen, The Netherlands

**Keywords:** catheter dysfunction, end-stage renal disease, removal, repositioning, urgent-start peritoneal dialysis

## Abstract

**Introduction:**

Urgent-start peritoneal dialysis (PD) in patients with newly diagnosed end-stage renal disease (ESRD) is a well-tolerated alternative to hemodialysis (HD). The primary aim of this study was to identify the demographic and clinical characteristics of ESRD patients, as well as the presurgical, surgical, and postsurgical factors associated with urgent-start PD complications.

**Methods:**

A retrospective cross-sectional observational study was performed on 102 patients with ESRD who merited urgent-start PD from January 2015 to June 2019. The primary clinical outcome measures were catheter leakage, dysfunction, and peritonitis, whereas the secondary outcomes were catheter removal, repositioning, and death. Statistical inferences were made with the χ^2^ or Fisher’s exact test and independent samples *t* tests.

**Results:**

One hundred two subjects (65 men, 63.7%) 56.2 ± 15.1 years old were included in this study; 64 of the subjects had diabetes and hypertension (62.7%). Catheter leakage occurred in 8 patients (7.8%), catheter dysfunction in 27 patients (26.5%), and peritonitis in 14 patients (13.7%); meanwhile, catheter removal occurred in 6 patients (5.9%), catheter repositioning in 21 patients (20.6%), and death in 3 patients (2.9%). Peritonitis was associated with younger age (i.e., 47.0 ± 16.8 vs. 57.6 ± 14.4 years; *P* = 0.014; 95% confidence interval [CI]: 2.2–19.1; odds ratio [OR] 0.96; *P* = 0.018; 95% CI: 0.92–099), higher creatinine levels upon admission (i.e., 20.2 ± 9.8 vs. 14.1 ± 8.3; *P* = 0.014; 95% CI: −10.9 to −1.2), and heart failure (OR 4.79; *P* = 0.043; 95% CI: 1.05–21.88). Patients with abdominal hernia were 7.5 times more likely to have their catheter leak (OR 7.5; *P* = 0.036; 95% CI: 1.14–49.54)*.* Catheter removal was associated with obesity (i.e., body mass index [BMI] of 31.6 ± 4.1 vs. 25.9 ± 4.9; *P* = 0.007; 95% CI: −9.8 to −1.6; OR 1.26; *P* = 0.013; 95% CI: 1.05–1.51) and Modification of Diet in Renal Disease glomerular filtration rate (MDRD-GFR) (i.e., 2.5 ± 0.6 vs. 3.7 ± 2.3; *P* = 0.003; 95% CI: 0.5–1.9).

**Conclusion:**

Peritonitis was associated with younger age, higher creatinine levels upon admission, and heart failure; meanwhile, catheter removal was linked to obesity and lower glomerular filtration rate. Compared with previous reports, our study included patients in which PD was initiated shortly after catheter insertion, making the intervention a true urgent-start PD. This study contributes to the existing urgent-start PD literature by providing evidence that urgent-start PD with catheter opening within 72 hours has limited complications, making it a relatively safe option.

## Graphical abstract

**Figure:**
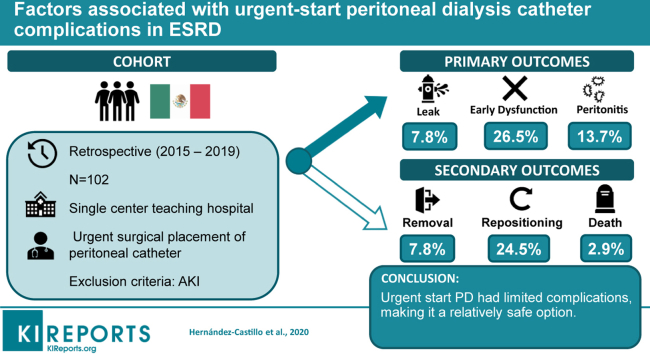

See Commentary on Page 1625

ESRD (i.e., stage 5 chronic kidney disease [CKD], estimated glomerular filtration rate <15 ml/min/1.73 m^2^) management can be divided into conservative and renal replacement therapy (RRT). RRT includes PD and HD. Urgent-start PD is warranted in patients with newly diagnosed ESRD who are not yet on dialysis and require RRT initiation within 2 weeks. Urgent-start PD is a feasible and well-tolerated alternative to HD.^1^ Adequate peritoneal catheter management is essential for the preservation of the peritoneal membrane function, thus permitting long-term PD management for CKD. However, urgent-start PD has shown an increased risk of leakage compared with routine or planned PD.^2^^,^^3^ Among the endogenous personal factors associated with peritoneal catheter dysfunction are morbid obesity, a history of abdominal surgeries, chronic abdominal wall infections, polycystic kidney disease, intestinal diseases, and severe malnutrition.^4^ Meanwhile, peritonitis is the primary exogenous factor associated with PD catheter failure, followed by exit-site leakage, surgical placement technique, and catheter migration.^4^^,^^5^ PD has been shown to prolong the length of RRT for patients with serious difficulties continuing with HD.^6^ Although the cost for RRT varies according to the health care system and reimbursement modalities,^7^ HD is consistently a more expensive option than PD.^8^ Furthermore, increased costs associated with HD could affect the patient’s therapeutic compliance, hence the importance of studying the factors that limit the function of PD.

We report a consecutive case series following the Strengthening the Reporting of Observational Studies in Epidemiology statement^9^ that includes 102 patients with CKD who merited urgent-start PD at a university teaching hospital. The primary aim of this study was to identify the demographic and clinical characteristics of ESRD patients, as well as the presurgical, surgical, and postsurgical factors associated with urgent-start PD complications. The complications (i.e., clinical outcome measures) investigated in this study were divided into primary and secondary outcome measures. The primary clinical outcomes were catheter leakage, catheter dysfunction, and peritonitis, whereas the secondary clinical outcome measures were catheter removal, catheter repositioning, and death. All outcome measures were associated with factors endogenous to the patient (i.e., demographic, CKD etiology, and clinical factors) and factors related to patient management (i.e., presurgical, surgical, and postsurgical factors). We present the occurrence of each type of complication and evaluate their association with the demographic, clinical, etiologic, and management factors of ESRD patients who merited urgent-start PD.

## Methods

A retrospective cross-sectional observational study was performed on 147 consecutive recruited patient medical files from individuals with CKD who merited RRT from January 2015 to June 2019 from the Department of Internal Medicine, Hospital General Dr. Manuel Gea González, Mexico City, Mexico. The inclusion criteria were age between 18 and 90 years, both sexes, diagnosis of CKD, presented urgently with CKD stage 5 without a plan for dialysis modality, met the National Institute for Health and Care Excellence criteria for RRT,^10^ had a PD catheter placed, were hospitalized at the internal medicine department, and were classified as urgent-start PD (i.e., initiation of PD in a patient with newly diagnosed end-stage kidney disease who were not on dialysis and who required dialysis initiation during the hospitalization within the first 96 hours). The exclusion criteria included PD catheter placement at a different hospital, hospital readmission for peritoneal catheter repositioning, and acute kidney injury. The elimination criteria included incomplete medical files and patient files without all outcome measures analyzed in this study. All patients were classified based on the 2012 Kidney Disease: Improving Global Outcomes guidelines^11^; thus, they had kidney damage (i.e., urinary albumin excretion of ≥30 mg and structural abnormalities detected by imaging) and decreased kidney function with an estimated glomerular filtration rate under 15 ml/min/1.73 m^2^, as well as 3 or more months of kidney disease irrespective of the cause.

The outcome measures of this study were catheter leakage, catheter dysfunction, peritonitis, and death. Catheter dysfunction was defined as a mechanical complication of internal and external prosthetic devices (i.e., *International Classification of Diseases, 10th Revision, Clinical Modification* code T85.6) that did not regain functionality after intracatheter interventions (i.e., heparin and fibrinolytic administration) or intestinal mobilization measures (e.g., the use of laxatives and walking). Catheter leakage was defined as an initial leakage of the intraperitoneal dialysis catheter (i.e., *International Classification of Diseases, 10th Revision, Clinical Modification* code T85.631A). Catheter removal (i.e., secondary to catheter mechanical breakdown initial encounter, *International Classification of Diseases, 10th Revision, Clinical Modification* code T85.611A) and catheter repositioning (i.e., secondary to catheter displacement, *International Classification of Diseases, 10th Revision, Clinical Modification* code T85.621, performed via an open surgical procedure) were secondary outcome measures resulting from dysfunction and catheter leakage. Peritonitis was defined clinically and through either a peritoneal lavage (i.e., positive for more than 100 cells or 50% of neutrophils and a positive peritoneal fluid culture) or positive bacterial culture of the removed catheter tip; additionally, peritonitis was determined within the first week of hospitalization. PD prescription was personalized for each patient considering fluid overload, uremia, and acid-base imbalance using an empirical approach. PD prescription was based on the International Society for Peritoneal Dialysis guidelines and the related literature.^12^ Briefly, for patients with primarily fluid retention, PD was initiated with a decremental approach initiated with a 4.25% dialysate solution down to a 2.5% glucose solution; meanwhile, for patients with primarily a uremic syndrome, a maximal prescription PD approach was indicated with 1.5% glucose solution. Dwell volumes ranged from 1 to 2 l (i.e., 10 patients had a dwell volume of 1 L, 23 of 1.5 l, and 69 of 2 l) and were chosen based on previous experience, taking into consideration the mean body surface area, which ranged from 1.53 to 1.80 m^2^. The median drain volume during the first 24 hours of postcatheter insertion was −350 ml (range −2020 to 1600 ml) for the patients with only fluid retention (*n* = 22), 100 ml (range −7630 to 1600 ml) for the patients with only uremic syndrome (*n* = 42), and −750 ml (range −5250 to 1080 ml) for patients with a combination of both conditions (*n* = 38); meanwhile, the final median drain volume was −6525 ml (range −22,925 to 3520 ml) for the patients with fluid retention, −200 ml (range −2945 to 6175 ml) for patients with only uremic syndrome, and −7870 ml (range −61,625 to 8500 ml) for patients with a combination of both conditions. Briefly, for patients with primarily fluid retention, PD was initiated with an incremental approach; however, for patients with primarily a uremic syndrome, a maximal prescription PD approach was indicated*.*

The predictive variables were divided into 2 categories, factors endogenous to the patient and factors associated with the management. Among the factors endogenous to the patient are sex, age, body mass index, previous abdominal surgery, presence of an abdominal hernia, CKD etiology (i.e., diabetes mellitus type 2, hypertension, chronic glomerulonephritis, polycystic kidney disease, and idiopathic), and different serum-derived biomarkers upon admission to the hospital (i.e., MDRD-GFR, albumin, creatinine, blood urea nitrogen, potassium, bicarbonate, and pH). The factors related to patient management were prophylactic antibiotic administration, administration of laxatives before the surgical procedure, the surgeon’s experience (i.e., years of surgical experience), and the time of catheter opening (i.e., the time between catheter placement and the start of PD). All variables of interest were extracted from medical files. Considering that the patients included are a convenience sample, consecutive medical files were included to reduce the risk of selection bias. The sample size was calculated based on a previous study investigating factors associated with PD catheter failure (i.e., 68% were assessed as clinical and technical factors in the evaluation of 149 catheters in 74 individuals, yielding a sample size needed of 87 for *n* = 149, α = 0.05, β = 0.8, *P* = 0.68 for overall catheter failure, and *P*_0_ = 0.55 for catheter dysfunction at 30 days or less) in a Mexican hospital.^13^ Upon hospital admission, the patients signed an informed consent permitting the use of their clinical file information for didactic, research, and publication purposes. This study was approved by the institutional review board of our hospital. Abiding by the Declaration of Helsinki, patient anonymity was guaranteed.

Statistical analysis was performed using SPSS 25 (IBM Corp, Armonk, NY). Data were screened for outliers and normality assumptions. The normality of continuous variables (i.e., time elapsed to peritoneal catheter dysfunction) was assessed with the Shapiro-Wilk normality test and visually using histograms and Q-Q plots. Dichotomous outcome variables such as catheter leakage, peritonitis, catheter removal, catheter repositioning, and death, as well as demographic, clinical, and management factors, are summarized using proportions and percentages. Categoric demographic variables (i.e., sex, previous abdominal surgery, and the presence of an abdominal hernia), as well as the categoric variable CKD etiology, were assessed for statistical inference with the χ^2^ or Fisher’s exact test. The clinical continuous variables (i.e., age, MDRD-GFR, serum creatinine, serum albumin, blood urea nitrogen, serum potassium, serum bicarbonate, and serum pH) were assessed for statistical inference individually using independent samples *t* tests and Levene's test for equality of variances to test for homoscedasticity. To assess the statistical inference for the factors related to patient management, a χ^2^ test was used for the presurgical, surgical, and 1 postsurgical factor (i.e., catheter opening time). Three multiple logistic regression models (i.e., demographic variables, clinical variables, and patient management variables) with backward stepwise elimination were performed for each primary outcome measure. An interaction term between MDRD-GFR and creatinine was added for the clinical model. Variables with multiple categories (i.e., CKD etiology, surgeon experience, and catheter opening time) were assessed for the difference between each categoric sublevel. The Omnibus Test of Model Coefficients was used to calculate overall model fitness and change between models. The –2 log-likelihood statistic (–2LL) was used to assess if the predictor contributed to the overall model. *R*_L_^2^ or Hosmer-Lemeshow *R*^2^ was computed using (–2LL_baseline_) – (–2LL_new_)/–2LL_baseline_; the Cox-Snell *R*^2^ and Nagelkerke *R*^2^ are also reported as effect sizes for the logistic regression model. Statistical significance was set at *P* ≤ 0.05.

## Results

One hundred forty-seven consecutive patients with CKD undergoing renal urgent-start replacement therapy with PD were identified. Forty-five patient files were excluded: 18 patients met the elimination criteria (i.e., had missing information or outcome measures), 15 did not meet the inclusion criteria (i.e., did not meet the age or National Institute for Health and Care Excellence criteria), and 12 met the exclusion criteria (i.e., catheter placement was in another hospital or had acute kidney injury). One hundred two patients were included in the analysis. The demographic characteristics of the included population were 65 male subjects (63.7%) with a median age of 56.2 ± 15.1 years (range 20–84 years), and the most common CKD etiology was the combination of diabetes mellitus type 2 and hypertension (64 patients, 62.7%); a complete demographic and clinical description of the patients is provided in Table 1. Presurgical, surgical, and postsurgical factors can be found in Table 2. In brief, the most common presurgical factor was antibiotic prophylaxis with 57 patients (55.9%); most catheters were placed by a surgeon with less than 2 years of experience (i.e., 41 surgeons with 1 year of experience and 44 with 2 years of experience), and the time between catheter placement and the start of peritoneal dialysis was commonly 24 hours (i.e., 79 cases, 77.5%). All patients underwent urgent-start PD within the first 96 hours. Specifically, 93 patients started PD within the first 24 hours, 7 between 24 and 48 hours, 1 at 72 hours, and 1 at 96 hours.

**Table 1:** Demographic and clinical characteristics of patients

**Demographic (N = 102)**	
Male	65 (63.7)
Age	56.2 ± 15.1 (20–84)
Body mass index	26.3 ± 5.1 (13.6–42.0)
Previous abdominal surgery	28 (27.5)
Abdominal hernia	6 (5.9)
**Etiology**	
Type 2 diabetes mellitus and hypertension	64 (62.7)
Hypertension	12 (11.8)
Type 2 diabetes mellitus	9 (8.8)
Idiopathic	7 (6.9)
Other	10 (9.8)
**Clinical**	
Modification of Diet in Renal Disease glomerular filtration rate, ml/min per 1.73 m^2^	3.6 ± 2.26 (0.85–12.06)
Albumin, mg/dl	2.5 ±0.6 (1.1–4.0)
Creatinine, mg/dl	15.0 ± 8.7 (1.5–39.0)
Blood urea nitrogen, mg/dl	132.1 ± 66.0 (16.6–342.0)
Potassium, mEq/l	6.2 ± 1.4 (2.8–9.2)
Bicarbonate, mEq/l	10.6 ± 4.3 (2.3–22.4)
pH	7.2 ± 0.2 (6.7–7.5)

Data are presented as *n* (%) or mean ± SD (range).

**Table 2:** Factors related to the patient management

**Presurgical factors**					
Antibiotic prophylaxis	57 (55.9)				
Laxative administration	17 (16.7)				
**Surgical factors**					
Surgeon’s experience, yr	1	2	3	4	5
*n*	43 (42.2)	44 (43.1)	8 (7.8)	5 (4.9)	2 (2)
**Postsurgical factors**					
Catheter opening time	Less than 24 h		More than 24 h		24 h
*n*	14 (13.7)		9 (8.8)		79 (77.5)
Time of hospitalization	12 days (Q2–Q3, 8.75–21.25; range 3–62)				

Data are presented as *n* (%) unless otherwise noted.

The primary clinical outcomes investigated in this study were catheter leakage, catheter dysfunction, and peritonitis. Catheter dysfunction happened in 27 cases (26.5%), 14 patients (13.7%) had peritonitis, and 8 patients (7.8%) had catheter leakage. No demographic, clinical, or management factors were associated with catheter leakage or catheter dysfunction upon the initial analysis. After performing the multivariate logistic regression models, patients with abdominal hernia were 7.5 times more likely to have their catheter leak (OR 7.5; *P* = 0.036; 95% CI: 1.14–49.54). Meanwhile, peritonitis was associated with the patient’s age (*P* = 0.014; 95% CI: 2.2–19.1) and serum creatinine levels at admission (*P* = .014; 95% CI, −10.9 to −1.2). Patients who had peritonitis were younger (i.e., 47.0 ± 16.8 vs. 57.6 ± 14.4; *P* = 0.014; 95% CI: −19.05 to −2.25; OR 0.96, *P* = 0.018; 95% CI: 0.92–0.99) and had higher creatinine levels upon admission (i.e., 20.2 ± 9.8 vs. 14.1 ± 8.3; *P* = 0.014; 95% CI: 1.24–10.92); however, MDRD-GFR was not associated with peritonitis (*P* = 0.119; 95% CI: −0.27 to 2.29). Patients with heart failure were almost 5 times more likely to have peritonitis (OR 4.79; *P* = 0.043; 95% CI: 1.05–21.88). Among the isolated bacteria were *Enterococcus faecalis*, *Proteus mirabillis*, *Pseudomonas aeruginosa*, *Staphylococcus aureus*, *Staphylococcus sciuri*, and *Morganella morganii*. Furthermore, 5 patients had an associated coinfection (i.e., 2 with pneumonia, 2 with exit-site infection, and 1 with bacteremia).

The secondary clinical outcomes were catheter removal, catheter repositioning, and death. Of the 27 patients who had catheter dysfunction, 21 patients had their catheters repositioned, and 6 patients had their catheter removed. Furthermore, of the 14 patients with peritonitis, 8 had a catheter dysfunction (i.e., 6 had their catheter repositioned, and 2 had it removed), 5 had hemodialysis, and 1 patient was deceased. Meanwhile, of the 8 patients with catheter leakage, 4 had their catheter repositioned, 2 had it removed, and 2 underwent hemodialysis. In total, 25 catheters were repositioned (24.5%) and 8 removed (7.8%). Stratification of the cases that had a catheter dysfunction allowed exploration of the possible associations between secondary clinical outcomes (i.e., catheter removal, catheter repositioning, and death) and the demographic, etiologic, clinical, and management factors. Catheter removal was associated with obesity (i.e., BMI of 31.6 ± 4.1 vs. 25.9 ± 4.9; *P* = .007; 95% CI: −9.8 to −1.6; OR 1.26; *P* = 0.013; 95% CI: 1.05–1.51) and lower MDRD-GFR (i.e., MDRD-GFR of 2.5 ± 0.6 vs. 3.7 ± 2.3; *P* = 0.003; 95% CI: 0.5–1.9). Only 3 patients (2.9%) were deceased, thus resulting in a lack of statistical power. Patient death was associated with sex (*P* = .045); however, all 3 patients were females. It is worth noting that 2 of the deceased patients had peritonitis, and the other one had the highest BMI in this study (i.e., 42.0 kg/m^2^). Frequencies of clinical outcomes and the statistical relationships between predictors and outcomes can be found in Supplementary Tables S1 and S2.

## Discussion

The primary aim of this study was to identify the demographic and clinical characteristics of CKD patients, as well as the presurgical, surgical, and postsurgical factors associated with urgent-start PD complications. This study contributes to the existing urgent-start PD literature by providing evidence of factors associated with catheter complications from an urban setting at a second-level hospital. Furthermore, it contributes to the existing evidence that urgent-start PD with the initiation of PD within 72 hours has limited complications, making it a relatively safe option. This case series is unique compared with the existing literature because the time between catheter insertion and the initiation of PD or catheter break-in time was relatively short (i.e., 24 hours or less in most cases), making the intervention a true urgent-start PD. Among the primary clinical outcomes, peritonitis was associated with younger age and higher creatinine levels upon admission, and patients with heart failure were almost 5 times more likely to have peritonitis; similarly, patients with an abdominal hernia were 7.5 more times likely to have catheter leakage than those who did not have an abdominal wall deficiency. Meanwhile, catheter removal was associated with obesity and MDRD-GFR; however, this association must be interpreted carefully because of the limited number of cases. Other complications in urgent-start PD catheters have been previously described in the literature. A small (*n* = 27) single-center nonrandomized study reported a higher rate of minor leaks in patients who were treated with urgent-start PD compared with non-urgent PD.^14^ Furthermore, leaks have been reported in urgent-start PD at a rate of 0% to 7.7%, 5% to 20%, and 0% to 33% depending on the surgical approach (i.e., open, laparoscopic, and percutaneous, respectively).^15^ Compared with elective-start PD, urgent-start PD had lower rates of catheter dysfunction (i.e., 17.2% vs. 28.4%) yet higher rates of catheter leakage (13.8% vs. 3.3%).^16^ The high number of leaks in this study could be associated with the short catheter break-in time. The time to catheter opening has also been proven to affect the frequency of complications. A short catheter break-in time increases the risk of leaks and probably is associated with pain (e.g., drain pain). In another study comparing patients with urgent-start PD (i.e., who started PD within 48 hours after catheter insertion vs. patients who began PD within 2 to 13 days) reported higher rates of technical complications in patients who began fluid infusion within 48 hours (i.e., 28.2% vs. 10.3%, *P* = 0.002).^14^ Similarly, catheter migration is also a complication associated with urgent-start PD and thus requires catheter repositioning or removal. Previously, catheter migration was associated with the surgical placement technique; open, laparoscopic, and percutaneous placement techniques were associated with rates of migration of 3.1% to 15.4%, 0% to 20%, and 6% to 20%, respectively.^15^ Furthermore, the need for catheter repositioning was higher in patients whose catheter break-in period was less than 48 hours (i.e., 14.6% vs. 3.4%, P = 0.009).^17^ In patients with continuous ambulatory PD, catheter break-in periods of less than 2 weeks are feasible and safe.^18^

Peritonitis was the only primary outcome measure associated with demographic and clinical factors in our study. Previously, peritonitis was the main factor (i.e., 84% of the cases) associated with late catheter dysfunction (i.e., more than 30 days after catheter placement).^4^ Furthermore, peritonitis has a hazard ratio for mortality of 1.47 (*P* = 0.001),^19^ hence the need to study peritonitis as a predictive factor for catheter dysfunction and death associated with longitudinal studies of urgent-start PD. Coinfections could play a role in complications associated with PD catheter placement. Although no patients had a tunnel infection, 2 patients with peritonitis had an exit-site infection. The retrospective nature of the study limits our ability to make associations with other factors that can be associated with peritonitis. We found that patients who had peritonitis were younger (i.e., 47.0 vs. 57.6 years; *P* = 0.014). Previous studies have reported mixed results regarding the association between peritonitis and age. Although older age has been linked to higher rates of peritonitis,^20^^,^^21^ other studies have reported no association^22^^,^^23^ or association with younger age.^24^ Our study also found that higher creatinine levels (20.2 vs. 14.1; *P* = 0.014) but not MDRD-GFR (*P* = 0.119) upon admission were associated with peritonitis. In this regard, Han *et al.*^25^ reported an association between reduced residual renal function (i.e., a glomerular filtration rate < 5 ml/min) and the risk of peritonitis but no association with creatinine levels.

The patient BMI has had mixed associations with peritoneal catheter dysfunction. Prasad *et al.*^26^ found an association between underweight (BMI < 18.5 kg/m^2^) and higher mortality, whereas obese patients (BMI > 25 kg/m^2^) had a greater risk for peritonitis; however, obesity (BMI > 30 kg/m^2^) had no association with catheter dysfunction.^27^ We identified a higher risk of catheter removal associated with obesity and lower MDRD-GFR. Although 1 of the 3 deceased patients had a BMI of 42.0 kg/m^2^, no association between and mortality peritonitis can be concluded from our study. A discussion about the selection of our dependent variables is also warranted. Two variables in our study could be interpreted as both clinical outcome measures and predictors. We decided to interpret peritonitis as a clinical outcome measure; however, the case can be made that peritonitis could be a predictor, especially for catheter removal and death. Our decision to interpret peritonitis as a clinical outcome measure was based on the study design.

### Limitations

A major limitation of this study is the size of the sample. We based our target sample size from work previously done in a clinical setting like ours. A small sample size poses the problem of overinterpretation of statistically significant results. To account for multiple comparisons, *post hoc* pair-wise comparisons with a Bonferroni correction were performed with *P* ≤ 0.0029 to account for the 17 independent variables evaluated in this study; however, no associations between clinical outcomes and the predictors survived the Bonferroni correction. Having a small sample size not only affects the reliability of statistical significances, but it also creates an inflationary bias in *R*2. To address the risk of having an interpretation bias secondary to an inflated *R*^2^, we report the *R*_L_^2^ (Hosmer-Lemeshow *R*^2^), the Cox-Snell *R*^2^, and Nagelkerke *R*^2^ to allow the reader to formulate their interpretation of the effect sizes derived from the logistic regression model. Furthermore, we abstain from the overinterpretation of these pseudo-*R*^2^ statistics. Another limitation related to the generalizability of this study is the time of assessment for the outcome measures. The outcome measures were only assessed during the time the patients were hospitalized, limiting the interpretation of the results to complications during an acute and subacute phase. Longitudinal assessment of predictors could provide more insight into long-term complications.

Catheter placement could be a contributing factor to catheter complications because most patients (i.e., 93 cases, 91.2%) had a medial catheter placement (i.e., under the umbilical scar). A paramedian placement site has been previously reported to have a low catheter migration rate and thus is considered safe.^28^ Furthermore, infused volumes greater than 500 ml and a medial placement site had a greater number of minor leakages.^14^ Most patients in this study fulfill both conditions because they were infused with volumes between 1000 and 2000 ml dialysis fluid (i.e., 10 cases with 1 L, 23 with 1.5 l, and 69 with 2 l). Some patients in this study had bridge hemodialysis before PD was initiated as well as hemodialysis as a management strategy for peritoneal catheter complications.

## Conclusion

This single-center retrospective study included 102 patients who underwent urgent-start PD. Endogenous and exogenous patient factors were assessed for their association with catheter leakage, catheter dysfunction, peritonitis, and death. Peritonitis was associated with younger age, higher creatinine levels upon admission, and heart failure; meanwhile, catheter removal was linked to obesity and lower MDRD-GFR. This study contributes to the existing urgent-start PD literature by providing evidence that true urgent-start PD with catheter opening within 72 hours has limited complications, making it a relatively safe option. However, larger prospective multicenter studies assessing these risk factors in urgent-start dialysis are needed.

## Disclosure

All the authors declared no competing interests.
