# Supplementary material for: Factors Associated With Urgent-Start Peritoneal Dialysis Catheter Complications in ESRD
Source: Kidney Int Rep. 2020 Jul 26;5(10):1722–8. doi: 10.1016/j.ekir.2020.07.025 (PMC7572310; doi:10.1016/j.ekir.2020.07.025)
Supplement: Supplementary File (PDF) [file mmc1.pdf]

Appendix

Supplementary Table 1. **Statistical relationships between predictors and clinical outcomes**

		Clinical outcomes					
		Primary			Secondary		
		Leakage	Dysfunction	Peritonitis	Removal	Repositioning	Death
	Frequency	8 (7.8%)	27 (26.5%)	14 (13.7%)	8 (7.8%)	25 (24.5%)	3 (2.9%)
**Demographical**	Sex	0.135	0.402	0.372	0.186	0.078	**0.045**
	Age	0.761	0.656	**0.014**	0.600	0.826	0.715
	BMI	0.302	0.274	0.616	**0.007**	0.705	0.070
	Previous abdominal surgery	0.210	0.478	0.196	0.527	0.173	0.622
	Abdominal hernia	0.069	0.504	0.598	0.688	0.641	0.832
**CKD etiology**		0.557	0.725	0.054	0.835	0.840	0.250
**Clinical**	MDRD-GRF	0.418	0.296	0.119	**0.003**	0.683	0.202
	Albumin	0.520	0.376	0.731	0.711	0.152	0.224
	Creatinine	0.653	0.909	**0.014**	0.886	0.921	0.951
	Blood urea nitrogen	0.448	0.772	0.220	0.886	0.630	0.677
	Potassium	0.918	0.907	0.356	0.923	0.605	0.241
	Bicarbonate	0.747	0.874	0.901	0.967	0.889	0.952
	pH	0.829	0.528	0.803	0.797	0.612	0.858
**Patient management**	Antibiotic prophylaxis	0.729	0.388	0.633	0.543	0.264	0.589
	Laxative administration	0.346	0.763	0.455	0.739	0.482	0.575
	Experience	0.695	0.564	0.299	0.860	0.916	0.575
	Opening time	0.455	0.549	0.128	0.395	0.593	0.154

CKD: chronic kidney disease. MDRD-GRF: Modification of Diet in Renal Disease glomerular filtration rate.

All reported p-values are uncorrected.

Supplementary Table 2. Logistic regression model coefficients and effect sizes

Model	Variable	χ^2	p	b	95% CI for Odds Ratio			Pseudo-R^2		
					Lower	Odds	Upper	H&L	C&S	Negelkerke
Peritonitis (n=14)										
Demographical	Age	5.59	0.018	-0.05	0.92	0.96	0.99	.067	.056	.102
Clinical	Heart failure	4.09	0.043	-2.063	1.05	4.79	21.88	.107	.071	.128
Catheter leakage (n=8)										
Demographical	Abdominal hernia	4.38	0.036	2.02	1.14	7.5	49.54	.071	.034	.081
Removal (n=8)										
Demographical	Body mass index	6.15	0.013	0.232	1.05	1.26	1.51	.111	.068	.189
Death (n=3)										
Clinical	MDRD-GRF	4.52	0.033	2.02	1.02	1.24	1.51	.071	.041	.176

CI: confidence interval. χ^2: Wald test. Beta value refers to the measure of the modeled effect that reflects the parameter estimate. All reported p-values are uncorrected. H&L: Hosmer & Lemeshow R^2. C&S: Cox & Snell R^2. MDRD-GRF: Modification of Diet in Renal Disease glomerular filtration rate.
